# Behavior of Olive Genotypes Against Quick Decline Syndrome (QDS) Caused by *Xylella fastidiosa* subsp. *pauca* in Apulia

**DOI:** 10.3390/plants14020157

**Published:** 2025-01-08

**Authors:** Mariangela Carlucci, Michele Antonio Savoia, Pompea Gabriella Lucchese, Valentina Fanelli, Isabella Mascio, Francesco Luigi Aurelio, Monica Marilena Miazzi, Andrea Pacifico, Cinzia Montemurro, Franco Nigro

**Affiliations:** 1 Department of Soil, Plant and Food Sciences (DiSSPA), University of Bari—Aldo Moro, Via Giovanni Amendola 165/A, 70126 Bari, Italy; mariangela.carlucci@uniba.it (M.C.); michele.savoia@uniba.it (M.A.S.); pompea.lucchese@uniba.it (P.G.L.); valentina.fanelli@uniba.it (V.F.); isabella.mascio@uniba.it (I.M.); francesco.aurelio@uniba.it (F.L.A.); monicamarilena.miazzi@uniba.it (M.M.M.); andrea.pacifico@uniba.it (A.P.); cinzia.montemurro@uniba.it (C.M.); 2 Spin Off Sinagri s.r.l., University of Bari Aldo Moro, Via Giovanni Amendola 165/A, 70126 Bari, Italy

**Keywords:** *Olea europea*, *Xylella fastidiosa*, resistance, tolerance, SSR, genotyping

## Abstract

*Xylella fastidiosa* subsp. *pauca* (*Xfp*), a quarantine pathogen in the European Union, severely threatens Mediterranean olive production, especially in southern Italy, where Olive Quick Decline Syndrome (OQDS) has devastated Apulian olive groves. This study addresses the urgent need to identify resistant olive genotypes by monitoring 16 potentially tolerant genotypes over six years, assessing symptom severity and bacterial load. These genotypes, which survived in heavily infected areas, showed varied responses to *Xfp*; some maintained low symptom severity with minimal bacterial presence (high or undetectable Cq values), while others exhibited increased bacterial loads yet remained asymptomatic or showed limited canopy desiccation. SSR markers were used to investigate the genetic relationships among these genotypes and other widespread Mediterranean cultivars, showing genetic similarity with the resistant ones such as the Albanian Kalinjot and the Greek Leucocarpa, as well as with local Apulian cultivars, highlighting the potential of local and Mediterranean olive germplasm for *Xfp* resistance. This study integrates phenotypic responses with genetic knowledge to support the development of conservation strategies that will enhance the genetic diversity of Apulian olive cultivars. In addition, by focusing on the resilience of the different olive genotypes, this research aims to protect the traditional cultivars from the emerging threats, thus preserving the ecological and cultural heritage of the olive biodiversity of the Mediterranean region.

## 1. Introduction

*Xylella fastidiosa* is a globally distributed, Gram-negative bacterium that poses a severe threat to agriculture, impacting a wide array of economically significant crops. More than 650 plant species, including olives, grapevines, almonds, citrus, and numerous ornamental plants are vulnerable to infection by various subspecies of this pathogen [1]. Transmission occurs via xylem sap-feeding insects, primarily sharpshooters (*Hemiptera*: *Cicadellidae*: *Cicadellinae*) and spittlebugs (*Hemiptera*: *Aphrophoridae*), which facilitate the bacterium’s inoculation into host plants. Upon entering the plant, *X. fastidiosa* colonizes the xylem vessels, obstructing water and nutrient transport, a disruption that severely compromises plant health [2,3].

Among the subspecies, *X. fastidiosa* subsp. *pauca* (*Xfp*) has emerged as particularly destructive, causing Olive Quick Decline Syndrome (OQDS), a disease responsible for the decimation of olive groves in Apulia, southern Italy (Latitude: 39.9500° N to 40.5500° N; Longitude: 17.5000° E to 18.5000° E). Since its first detection in 2013 [4], the epidemic has spread across extensive areas, leading to substantial economic losses in one of Italy’s key olive-producing regions and profoundly affecting the region’s landscape and socio-economic conditions [5,6].

The clinical manifestations of OQDS include leaf scorch, progressive twig and branch desiccation, and ultimately, canopy dieback, which can culminate in tree death [7]. These symptoms are primarily attributed to the occlusion of xylem vessels by bacterial aggregates encased in an exopolysaccharide matrix. This blockage, compounded by the formation of tyloses and gums as part of the plant’s immune response, further disrupts water flow within the xylem [8]. The severity of OQDS underscores the urgent need for integrated management strategies, including genetic research, vector control, and plant health monitoring, to mitigate the impact of *Xfp* on Mediterranean agriculture and preserve the biodiversity and productivity of affected regions.

The detection of *Xylella fastidiosa* subsp. *pauca* (*Xfp*) on olive trees in Apulia, southern Italy, marked a pivotal moment for the region’s agricultural sector, severely impacting the local economy, which heavily depends on olive and olive oil production and trade [9,10]. *Xfp* has been designated as a quarantine and priority pest under EU Regulations 2019/1702 and 2020/1201 [11,12], mandating compulsory surveillance programs across all member states. Legislative measures for managing this pathogen include strict eradication and containment strategies to limit the spread of infections and reduce inoculum pressure, thereby aiming to mitigate the disease’s detrimental effects.

Managing *Xfp* is particularly challenging due to its wide host range, the rapid transmission by insect vectors, and the absence of effective curative treatments. Current control strategy primarily concentrates on halting pathogen spread through rigorous infection monitoring, the removal of infected plants, and replanting with resistant or tolerant genotypes [13,14,15]. The scale of the outbreak underscores the necessity of developing integrated pest management strategies that incorporate vector control, the use of genetic research to identify and propagate resilient cultivars, and enhanced biosecurity measures to safeguard the biodiversity and economic stability of olive-growing regions.

In situations where eradication is mandated or compulsory, replanting with resistant or tolerant olive genotypes stand out as the most viable and sustainable strategy to control *Xfp* and minimize its impact. The concepts of resistance and tolerance to diseases are critical for understanding how plants cope with pathogens. Resistance refers to the ability of a plant to prevent or limit the establishment and spread of pathogens, effectively reducing the severity of disease symptoms. This can occur through various mechanisms, including physical barriers, such as thickened cell walls, and biochemical responses, such as the production of antimicrobial compounds [16,17]. On the other hand, tolerance is defined as the capacity of a plant to sustain damage caused by pathogens without a significant loss in fitness or reproductive success. Tolerant plants may allow pathogen growth but possess traits that mitigate the negative impacts of the infection, such as enhanced growth rates or compensatory mechanisms that promote recovery [18,19]. Notably, the Leccino and FS17 (Favolosa) cultivars have exhibited significant resistance and tolerance traits, respectively, characterized by lower infection rates and reduced symptom severity compared to susceptible cultivars such as Ogliarola Salentina and Cellina di Nardò [20,21]. The behavior observed in these genotypes is due to a combination of defense mechanisms, encompassing biochemical responses and anatomical adaptations that collectively enhance resilience against *Xfp* [20,22,23,24]. Recent studies have shown that in the cv Leccino resistance is regulated by multiple gene pathways, including heightened activity of cell-wall-associated protein kinases in infected tissues. Furthermore, distinct gene expression profiles related to photosynthesis and metabolic processes have been observed in *Leccino* genotypes with spontaneous resistance traits, highlighting the genetic complexity of pathogen resistance [25]. Conversely, the cultivar Fs-17 (Favolosa) is recognized as a tolerant cultivar. Although it allows bacterial colonization, it exhibits reduced symptom severity and slower disease progression compared to highly susceptible cultivars like Ogliarola Salentina and Cellina di Nardò. Fs17 maintain functional xylem vessels despite pathogen colonization and show lower levels of symptom severity, such as leaf scorch and canopy desiccation. Moreover, it sustains productivity over longer periods compared to susceptible cultivars [24]. 

These findings underscore the importance of ongoing genetic research and breeding programs aimed at enhancing resistance in olive cultivars. By identifying and propagating resilient genotypes, such initiatives could play a crucial role in mitigating the spread of *Xylella* infections and ensuring the long-term sustainability of olive production. Therefore, it is essential to continue the screening of the olive biodiversity to identify additional genotypes that can be included in breeding programs and used for disease management [26,27].

Traditional morphological characterization, which is highly susceptible to environmental influences [28], has been fundamental to olive classification and is now supported by molecular markers, such as simple sequence repeats (SSRs) and single nucleotide polymorphisms (SNPs), which allow more accurate genetic indexing of olive cultivars [29,30,31]. Microsatellite markers, or simple sequence repeats (SSRs), are characterized by high polymorphism, making them particularly useful in organisms where alternative markers provide limited information. In olive research, SSRs are advantageous because of codominant inheritance, high reproducibility, interspecies transferability, and cost-effectiveness [32].

DNA-based analytical techniques can provide precious information on genetic diversity, especially of Apulian olive germplasm, and on resistance traits to *Xfp*. The genetic knowledge of resistant or tolerant olive trees can contribute to the resilience and sustainability of olive production in *Xfp*-affected regions by helping to develop robust selection programs that incorporate different resistance mechanisms. 

The present study aimed to identify olive genotypes that have survived the *Xfp* epidemic in Apulia, suggesting they may harbor resistance or tolerance traits against this destructive pathogen. Specifically, this research sought to (i) monitor the progression and severity of symptoms in olive genotypes presumed to exhibit resistance or tolerance, (ii) detect and quantify the presence of *Xfp* in these plants using sensitive molecular assays, and (iii) evaluate the genetic relationships between these resilient genotypes and a comprehensive panel of 244 Mediterranean olive cultivars. By elucidating both the phenotypic responses and genetic relationships, this study aims to support the development of targeted breeding programs and conservation strategies that bolster the genetic resilience and biodiversity of Apulian olive populations affected by *Xfp*.

Moreover, this work contributes to ongoing efforts to safeguard the diverse genetic heritage of olive cultivars in Apulia, ensuring the preservation of the region’s rich biodiversity. By focusing on the unique traits and resilience of various olive genotypes, this research supports strategies aimed at protecting traditional cultivars from emerging threats, thus preserving the ecological and cultural legacy of Mediterranean olive biodiversity. The outcomes of this study will not only aid local olive production but also offer valuable insights for other regions facing similar pathogen pressures, promoting a proactive approach to preserving global olive biodiversity in the face of agricultural challenges.

## 2. Material and Methods 

### 2.1. Field Survey for Identification of Surviving Genotypes

Extensive surveys were conducted in heavily infected olive-growing areas within the provinces of Brindisi and Lecce (Apulia, southern Italy), where OQDS has resulted in the death of at least 95% of olive trees. Beginning in 2018, olive trees that survived the epidemic—ranging from 30 to 40 years in age—were selected based on their remarkable vegetative condition and the presence of minimal or no disease symptoms. These trees were georeferenced to enable continuous monitoring and further research. In total, 16 olive genotypes were identified (Table 1). 

The genotype P1_A, characterized by promising agronomic traits such as early ripening and a dwarf, compact habitus, was grafted onto a highly susceptible plant of the cultivar *Ogliarola Salentina*, which exhibited severe OQDS symptoms. This grafting combination, designated as P1_B, was analyzed separately to evaluate potential variations in infection dynamics and resilience when paired with a cultivar highly susceptible to *Xfp*. The goal of this approach was to determine whether the favorable traits of P1_A, particularly its resilience, could be influenced by the susceptible and severely affected rootstock.

Over the past 3 to 6 years, all selected genotypes have been monitored for *Xfp* bacterial load and symptom progression, as outlined below. Symptom severity scoring and sample collection for molecular diagnostic testing were conducted twice yearly, in April and October. To ensure consistency in data reporting, only the highest symptom severity score and the least favorable diagnostic test result from the two assessments were included in this report (Table 1 and Table 2).

### 2.2. Assessment of OQDS Symptoms Severity 

To assess the severity of OQDS symptoms, each selected tree was visually inspected using descriptive keys tailored to disease severity, enabling precise measurement and interpretation of the symptomatic percentage of the canopy. Symptom severity was evaluated according to a well-established empirical scale, rating symptoms from 0 to 5 as follows: 0 = no visible symptoms; 1 = symptoms limited to one or a few isolated twigs (less than 10% of the canopy affected); 2 = symptoms present on multiple twigs or a single branch (11–40% of the canopy affected); 3 = symptoms affecting several branches (41–60% of the canopy affected); 4 = extensive symptoms across the canopy (61–85% of the canopy affected); and 5 = severe symptoms, including branch dieback and overall tree decline (more than 86% of the canopy affected) [14].

### 2.3. Diagnostic Test and Estimation of Xfp Population in the Olive Tree Tissue

Sampling of plant material was performed systematically, with twigs and leaves collected from the four cardinal points around each tree canopy, carefully avoiding tissues in advanced stages of desiccation to ensure accurate assessment of bacterial load. Samples were tested for *Xfp* infection on the same day as collection, following the standard EPPO protocol (PM 7/24(5)) [33]. Total DNA extraction was carried out on 0.5 g of petioles collected from mature leaves, which were placed in a sealed sterile bag (BIOREBA AG, Switzerland) containing 5 mL of CTAB buffer (2% hexadecyltrimethylammonium bromide, 0.1M Tris-HCl pH 8, 20 mM EDTA, 1.4 M NaCl, and 1% PVP-40) and homogenized using a Homex homogenizer (BIOREBA AG, Switzerland). The samples were then processed for total DNA extraction [34], with an additional treatment of 50 µg/mL RNase A (Zymo Research Corporation, Orange, CA, USA). The presence of *Xylella fastidiosa* was assessed via quantitative polymerase chain reaction (qPCR) according to the protocol described by Harper et al. [35], using the primers XF-F (5′-CACGGCTGGTAACGGAAGA-3′), XF-R (5′-GGGTTGCGTGGTGAAATCAAG-3′), and the probe XF-P (5′-FAM-TCGCATCCCGTGGCTCAGTCC-BHQ1-3′). Reactions were performed with 100 ng/µL of total DNA. The qPCR reactions were carried out on a CFX 96™ Real-Time System using SsoAdvanced Universal Supermixes (BioRad Laboratories, Hercules, CA, USA) under the following conditions: an initial denaturation at 95 °C for 5 min, followed by 40 cycles of 94 °C for 10 s and 62 °C for 40 s. The estimated *Xfp* population size, corresponding to each threshold cycle value (Cq), was inferred from a standard calibration curve. This curve was derived from a triplicate assay using DNA extracted from 10-fold serial dilutions of bacterial suspensions, ranging from 10^7^ to 10^2^ CFU/mL, spiked into homogenized tissues of non-infected olive samples [36].

### 2.4. Olive Genotyping Using SSR Markers

Genomic DNA for plant genotyping was extracted from young, healthy leaves of the 16 olive trees using the protocol described by Spadoni et al. [37]. The quality and concentration of the DNA were evaluated via 0.8% agarose gel electrophoresis and quantified using a NanoDrop™ ND2000c spectrophotometer (Thermo Scientific, Waltham, MA, USA).

Genotyping was carried out using a set of nine SSR markers (Supplementary Table S1) that are widely employed for studying genetic variability in olives [37,38,39]. These markers were selected based on their reliable amplification, high polymorphism, and reproducibility [30].

PCR reactions were carried out in a final volume of 12.5 µL, as described by Miazzi et al. [40]. The PCR products were then separated via capillary electrophoresis using an ABI PRISM 3100 Avant Genetic Analyzer (Applied Biosystems, Foster City, CA, USA), with GeneScan 600 LIZ serving as the reference size standard (Applied Biosystems, Foster City, CA, USA). Allelic sizes of the amplicons were estimated using GeneMapper v.5.0 software (Applied Biosystems, Foster City, CA, USA).

The molecular profiles obtained were compared with those of 244 Italian and international olive accessions stored in the database of the Department of Soil, Plant, and Food Sciences (Di.S.S.P.A.) at the University of Bari, Italy. This database includes resistant varieties, such as Leccino and FS17, as well as susceptible cultivars like Cellina di Nardò and Ogliarola Salentina. Additionally, molecular profiles of five olive genotypes—Secolare di Chieuti, Leccio del Corno, Leccino Lazio, Spina, and Dolce Tonda—identified as potentially resistant in a study by Savoia et al. [14], were incorporated into the dataset.

Pairwise relationship analysis (LRM) [41] was performed to assess allelic similarity and identify potential synonymies using GenAlEx v.6.502 software [42]. The paternity analysis was conducted using Cervus v.3.0 software to identify potential parentage among the 16 selected samples. Finally, phylogenetic relationships among the olive samples and database accessions were analyzed through the construction of a Neighbor-Joining tree [43] with Darwin5 v.6.0.010 software (http://darwin.cirad.fr, accessed on 4 October, 2024). The robustness of the tree branches was evaluated with 1000 bootstrap replicates [44]. 

## 3. Results

### Field Survey for Identification of Surviving Genotypes and Assessment of Symptoms Severity and Bacterial Load

The field survey and the assessment of symptom severity caused by *Xfp* in infected areas, led to the identification of 16 olive genotypes that survived the epidemic exhibiting low or no symptomatology. These plants were monitored for symptom severity and tested for pathogen quantification from 2019 to 2024 (Table 1 and Table 2). The data in Table 1 and Table 2 reveal insights into symptom severity, threshold cycle (Cq) values, and estimated bacterial populations (CFU/mL) respectively in various olive genotypes over a six-year period (2019–2024). At the initial symptom assessment in 2019, the 16 selected olive genotypes were either asymptomatic or exhibited low symptom severity, with high or undetectable Cq values (U). In 2020, this condition remained stable, except for P14, which showed an increase in symptom severity and a Cq value of 31.37.

Analysis of symptom trends and bacterial concentrations for individual genotypes in subsequent observation years revealed two distinct patterns of disease response: (1) genotypes with consistently low bacterial loads (high or undetectable Cq values) and minimal symptoms (severity between 0-1), including P1_A, P3_Sud, P3_Nord, P6-P12, and P14; and (2) genotypes initially displaying low symptom severity, followed by an increase in bacterial load and symptom severity in the sixth year of observation (P2 and P13).

Infection data for samples P15, P16, and P17 from 2019 to 2023 were unavailable; however, in 2024, P15 and P16 exhibited high bacterial loads with Cq values of 20.46 and 21.15, respectively, and a symptom severity of 1. Genotype P17 remained asymptomatic and showed a low bacterial concentration (Cq value of 35.19) in 2024. Notably, genotype P1_B showed an increase in symptoms and bacterial load in 2021, followed by a return to asymptomatic status and reduced bacterial load in subsequent years.

In several genotypes, such as P1_A, P1_B, and P3_Nord, symptom severity fluctuated, often correlating with detectable Cq values and estimated bacterial populations. For instance, P1_A showed symptoms in 2019, 2020, and 2024, with Cq values indicating bacterial presence, but had undetectable levels in 2021 and 2023, suggesting intermittent pathogen detection or response. In contrast, genotype P10 consistently displayed low symptom severity (SS = 1) and detectable bacterial loads each year, suggesting a different behavior to infection. Some genotypes, like P7, P8, and P12, had undetectable bacterial levels throughout the study, indicating possible resistance/tolerance or effective suppression of bacterial populations. The genotypes P15, P16, and P17 were identified and included in the comparison just in 2024 but showed the highest bacterial counts, reaching up to 10^7^ CFU/mL, with very low symptoms severity.

The samples were molecularly characterized by nine olive-specific microsatellite markers showing a clear allelic profile. The results of the LRM analysis, based on pairwise comparisons of the analyzed samples, are shown in Table 3. Samples P1_A and P1_B were identical (LRM value of 0.5) and originated from the same genotypes, while the other comparisons highlighted high genetic similarity (LRM values included between 0.25 to 0.48) between some samples.

Then, the comparison was carried out between the analyzed olive genotypes and the accessions available in the Di.S.S.P.A. database showing for seven samples a high genetic similarity (LRM value > 0.25) with some Italian and European accessions (Table 3). Specifically, P6, P7, P12, P15, P16, and P17 showed a high genetic similarity with the Italian cultivars Ciciariello, Pendolino, Leccino, Taggiasca, Ascolana, Tenera, and Cipressino, respectively, while the genotype P13 resulted to be similar to the Albanian cultivar Kalinjot oval. 

The paternity analysis, performed on the tested genotypes, identified at least one putative parental for seven genotypes (Table 3). Leccino was detected as a putative parent of samples P13 and P14. The Albanian cultivar Kalinjot Oval was identified as a putative parent of genotypes P13 and P2. Genotypes P6 and P16 were putatively derived from the Greek cultivar Leucocarpa and the Algerian cultivar Aayrouni, respectively. Finally, the cultivars Pendolino and Framichele were identified as putative parents of genotypes P7 and P8, respectively.

**Figure 1:**
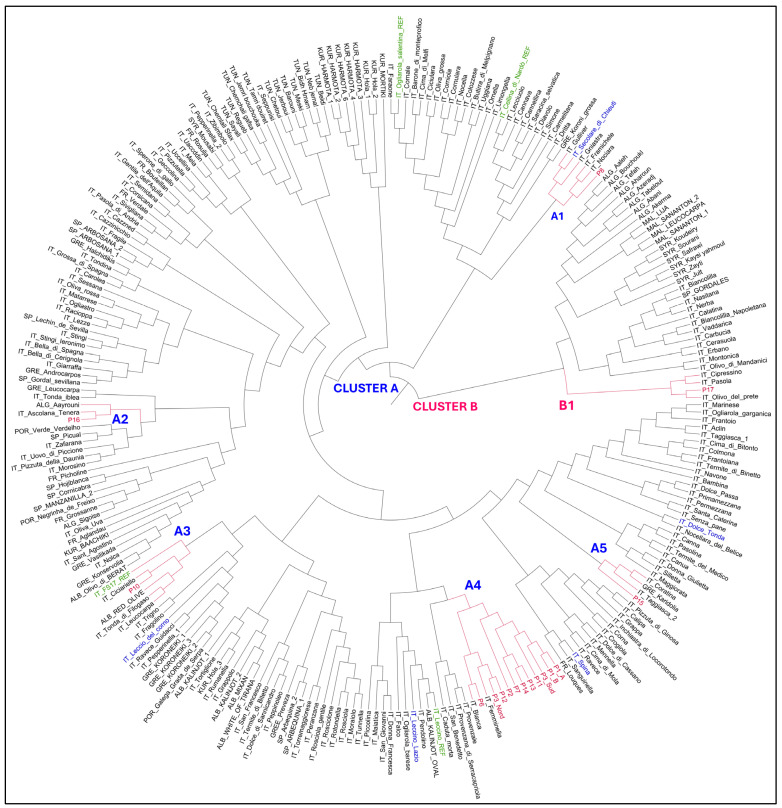
Neighbor-joining tree showing the phylogenetic relationships among 16 putatively resistant genotypes (in red) and 244 Italian and international olive accessions. Reference resistant and susceptible varieties are indicated in green, and the putatively resistant genotypes (PR) identified by Savoia et al. [14] are shown in blue.

The Neighbor-joining tree grouped the olive genotypes into two groups; the main group, Cluster A, included 226 genotypes, and the second one, Cluster B, consisted of 34 genotypes (Figure 1). All olive genotypes putatively resistant to *Xfp* were included in cluster A, except for sample P17 belonging to subcluster B1 together with some Italian cultivars. Cluster A included five subclusters (A1-A5). Subcluster A1 comprised the P8 genotype along with some Italian cultivars, such as Secolare di Chieuti found putatively resistant to *Xfp* by Savoia et al., 2023. Subcluster A2 included sample P16 along with the Italian Ascolana tenera and the Algerian Aayrouni. Genotype P10 was included in subcluster A3 together with some Italian cultivars and the Albanian Red Olive. This subcluster was found to be genetically related to the resistant variety FS17. Most of the analyzed olive genotypes (P1A, P1B, P2, P3 South, P3 North, P6, P7, P11, P12, P13, and P14) were included in subcluster A4 showing a high genetic similarity. Subcluster A4 belonged to a larger cluster containing the resistant variety Leccino. Finally, genotype P15 belonged to subcluster A5 together with the Italian cultivars Taggiasca and Coratina and the Greek Karidolia.

## 4. Discussion

The outbreak of *Xylella fastidiosa* subsp. *pauca* (*Xfp*) was first reported in 2013 in Salento, Apulia, Italy, and has since caused significant devastation, infecting approximately 22 million olive trees [6,7]. Despite efforts such as monitoring, containment zones, and removal of infected plants, these measures have only partially mitigated the epidemic and failed to eradicate the pathogen [45]. Consequently, adopting long-term strategies, including the replanting of resistant or tolerant genotypes, has become crucial. This study focuses on identifying olive genotypes with resistance or tolerance to *Xfp* by analyzing trees that survived the epidemic. The distinction between resistance and tolerance is essential for plant breeding and management strategies. Understanding the genetic architecture of disease resistance and tolerance is crucial for developing effective breeding programs aimed at enhancing these traits in crops [17]. Furthermore, the epidemiological consequences of deploying resistant versus tolerant varieties can significantly influence agricultural practices and pest management strategies [46]. Resistant varieties may reduce disease prevalence in a population, while tolerant varieties may allow for the coexistence of pathogens, potentially leading to different dynamics in disease spread and management.

Evaluating local germplasm and wild olive accessions is crucial for discovering new resistance or tolerance sources, aiding sustainable olive production [22,40,47]. The study examined genotype-specific responses to *Xfp* by tracking infection dynamics, symptom severity, and bacterial loads using qPCR under diverse conditions.

Given the time and costs involved in establishing controlled experimental olive groves to screen germplasm responses to *Xfp*, this study adopted a field-based approach.

The phenotypic response to *Xfp* and bacterial population estimates of 16 selected olive genotypes were monitored over a 6-year period. Cq values remained above 24, except in samples P13, P15, and P16, which exhibited increased bacterial populations in the sixth year. Sample P1_B showed an increase in both symptom severity and bacterial population in the third year (2021), followed by a return to lower infection levels in subsequent years. The analyzed samples displayed bacterial populations that did not consistently align with symptom severity, as values ranging from 2.43 × 10^2^ to 2.35 × 10^7^ cells/mL were associated with symptom severity never exceeding 1.3, which corresponds to 11–40% canopy desiccation. Overall, these data underscore substantial variability in bacterial population dynamics and symptom expression across genotypes, indicating differing degrees of susceptibility and potential resilience [48]. These findings highlight the importance of monitoring both symptom severity and bacterial loads to accurately assess genotype behavior towards the disease. The low infection rates observed in the tested genotypes could have been influenced by various epidemiological factors, such as vector population dynamics and the effects of single versus multiple infection events. Therefore, pathogen density and other environmental stressors can affect resistance or tolerance expression. It has been reported that the initial density of pathogens can significantly impact the expression of both resistance and tolerance in *Arabidopsis thaliana*, suggesting that these traits may interact in nuanced ways during plant-pathogen interactions [49]. Furthermore, the deployment of resistant versus tolerant varieties can have distinct epidemiological consequences, affecting not only individual plant health but also the dynamics of pathogen populations within agricultural systems [46].

Moreover, the mechanisms underlying resistance or tolerance of certain olive cultivars to *Xfp* are multifaceted, involving structural, biochemical, and microbiological factors. Research indicates that resistance and tolerance can coexist within the same plant species, allowing for a more nuanced understanding of plant defense strategies [50,51]. Anatomical characteristics of xylem vessels in different olive cultivars have been shown to influence susceptibility to *Xfp*. An image-based study revealed significant differences in xylem geometry between resistant and susceptible olive cultivars, suggesting that structural traits may play a role in limiting pathogen spread and reducing symptom severity [52]. These findings indicate that metabolic pathways related to stress responses, secondary metabolite production, and nutrient allocation play a central role in determining the susceptibility of the tested olive genotype to the pathogen.

Furthermore, understanding interactions between the tested olive genotypes and their associated microbiomes may lead to innovative approaches in biocontrol and integrated pest management. For example, Vergine et al. [53] demonstrated the role of the endophytic microbiota in the resistant cultivar Leccino, suggesting that beneficial microorganisms may contribute to the plant’s defenses against the pathogen. The stability of the endophytic community in resistant cultivars could bolster resilience by promoting plant health and mitigating the impact of pathogenic infections [54].

LRM analysis revealed genetic identity between samples P1_A and P1_B, as expected given their origin from the same grafted source. These accessions, along with samples P2, P3_South, P3_North, P7, and P11-P14, exhibited high genetic similarity, suggesting a shared genetic basis among these putatively resistant genotypes (Table 3). Despite the genetic identity observed between P1_A and P1_B in the LRM analysis, the accessions displayed different responses to the disease, indicating that various factors can influence susceptibility or resistance to the pathogen. Given that P1_B shares the same genotype as P1_A but is grafted onto the highly susceptible cv. Ogliarola salentina, it can be hypothesized that, in addition to genotype-environment interactions, the grafting combination may also impact the susceptibility or resistance response to the pathogen. Studying this graft combinations could aid in understanding the role of genotype interactions in disease resistance, thereby informing future grafting practices and breeding strategies to enhance olive resilience against OQDS.

When comparing the allelic profiles of sampled genotypes with the Di.S.S.P.A. dataset, sample P12 showed genetic similarity to the resistant cultivar Leccino, which may explain this plant’s ability to withstand the disease (Table 3). Among other cultivars showing similarity to the sampled accessions, moderate susceptibility has been documented for cultivars Cipressino and Pendolino [14,25]; however, resistance or susceptibility to *Xfp* remains undocumented for the other cultivars. Examining their responses to the pathogen may provide valuable insights into the mechanisms underlying resistance. 

Paternity analysis provided insights into the putative parents of seven sampled genotypes (P2, P6, P7, P8, P13, P14, and P16). Interestingly, some of the identified parent cultivars appear to originate from Albania, Algeria, and Greece. This is consistent with previous observations of diverse gene pools within Italian olive germplasm, originating from different regions of the Mediterranean Basin [55]. The presence of foreign cultivars among the parentage of Apulian accessions may be due to the extensive consumption of olive drupes by migratory birds, along with wind pollination, which likely facilitated long-distance seed dispersal [56,57]). This ecological process is essential for maintaining biodiversity within agricultural landscapes [58]. Examining olive germplasm from various Mediterranean countries may reveal valuable traits, including resistance to *Xfp*.

Of particular interest is the putative parentage of sample P6, attributed to cultivars Bianca and Leucocarpa, both highly susceptible but integral to the Apulian olive-growing tradition [14,59]. The resistance observed in the P6 genotype could be due to genetic recombination in progeny from two susceptible parents. There is precedence for resistant cultivars derived from susceptible parents, as seen in FS17, which descends from the partially susceptible Frantoio cultivar [60,61].

The phylogenetic tree revealed considerable genetic variability among the analyzed samples. As anticipated, some samples exhibited genetic similarity to Leccino and FS17. Notably, accession P8 showed a high degree of similarity to Secolare di Chieuti, identified as putatively resistant by Savoia et al. [14]. The identification of new resistance sources beyond the well-known Leccino and FS17 provides a valuable resource for replanting infected areas and preserving local biodiversity. Additionally, recent findings indicating that Leccino displays symptoms of disease [14,34] underscore the importance of identifying a broader range of resistant genotypes. The comprehensive sampling strategy adopted in this research allowed for an effective evaluation of symptoms severity and *Xfp* population dynamics within each tree, contributing to a detailed understanding of host-pathogen interactions. By identifying and analyzing trees that exhibit strong vegetative vigor and minimal or no symptoms of quick decline syndrome in naturally infected areas, this study leverages natural selection to facilitate the identification of resistant or tolerant genotypes. Based on the results, it can be hypothesized that genotypes P1_A, P2, P3 Nord, P10, P11, P13, and P14 exhibit a tolerant behavior, while genotypes P7, P8, and P12 appear to possess resistance traits. However, further testing via artificial inoculation of rooted cuttings with precisely defined pathogen concentrations is necessary to confirm their resistant or tolerant behavior.

Screening olive biodiversity is a critical step in identifying genotypes with potential resistance to *Xfp*, which could significantly contribute to breeding programs and conservation initiatives. Understanding and leveraging genetic resistance among olive cultivars provides a foundational basis for selecting resilient genotypes, which are essential for long-term crop stability. By integrating this genetic knowledge with optimized agronomic practices, it is possible to develop comprehensive, sustainable management strategies to mitigate the impact of OQDS on olive production. Such an approach not only enhances disease resilience but also supports the conservation of olive genetic diversity, ultimately contributing to the adaptability and health of olive agroecosystems. 

The samples considered in this study are promising as a source of *Xfp* resistance, considering that the symptomatology remained limited to a few twigs even where an increase in the bacterial population was observed. Future research on these putatively resistant genotypes will facilitate a clearer understanding of their mechanisms of resistance or tolerance to *Xfp*. The genetic resistance or tolerance of olive trees to *Xfp* is a complex trait influenced by multiple genetic and environmental factors. Identifying the specific genes and metabolic pathways involved in resistance is essential for developing effective breeding strategies. Leveraging the genetic diversity of olive cultivars and employing advanced breeding techniques will enhance the resilience of olive trees against this devastating pathogen, ensuring the sustainability of olive production in affected regions. 

Moreover, future research should further investigate the influence of vector population density and behavior on infection rates, as well as the role of single versus multiple inoculations in disease progression.

## Figures and Tables

**Table 1:** Symptom severity across the years.

Genotypes	Symptom Severity					
	2019	2020	2021	2022	2023	2024
P1_A	1	1	0	0	0	1
P1_B	0	0	1,4	0	1	0
P2	0	0	1	0	1	1,3
P3_Sud	0	0	1	0	1,5	1
P3_Nord	1	1	1	0	1	1
P6	-	-	-	1	0	1
P7	1	0	0	0	0	0
P8	1	0	0	0	0	0
P10	1	1	1	1	1	1
P11	1	1	0	0	0	1
P12	0	0	0	0	0	0
P13	1	1	1	0	1	1
P14	0	1	0	1	1	1
P15	-	-	-	-	-	1
P16	-	-	-	-	-	1
P17	-	-	-	-	-	0

- Indicates missing data, because the genotypes were identified later.

**Table 2:** Threshold cycle values and estimated bacterial population (Cfu/mL) in the tested genotypes.

Genotypes	2019		2020		2021		2022		2023		2024	
	Cq	Cfu/mL	Cq	Cfu/mL	Cq	Cfu/mL	Cq	Cfu/mL	Cq	Cfu/mL	Cq	Cfu/mL
P1_A	35.87	4.5 × 10^3^	34.66	3.2 × 10^3^	U	U	35.67	4.24 × 10^3^	U	U	34.22	3.52 × 10^3^
P1_B	U	U	U	U	24.78	3.12 × 10^6^	U	U	U	U	37.24	8.58 × 10^2^
P2	U	U	U	U	31.77	4.35 × 10^4^	U	U	36.63	1.55 × 10^3^	27.53	2.43 × 10^5^
P3_Sud	U	U	U	U	30.58	5.71 × 10^4^	U	U	28.40	2.77 × 10^5^	32.68	9.61 × 10^3^
P3_Nord	33.40	6.8 × 10^4^	U	U	34.87	2.97 × 10^3^	35.67	1.27 × 10^3^	30.33	8.10 × 10^4^	U	U
P6	NA	NA	NA	NA	NA	NA	35.77	1.72 × 10^3^	37.24	2.43 × 10^2^	U	U
P7	U	U	U	U	U	U	U	U	U	U	U	U
P8	U	U	U	U	U	U	U	U	U	U	U	U
P10	32.13	7.5 × 10^4^	32.24	7.1 × 10^4^	31.46	1.24 × 10^4^	29.45	3.65 × 10^5^	31.39	9.4 × 10^4^	U	U
P11	34.70	4.6 × 10^3^	35.67	1.4 × 10^3^	U	U	U	U	U	U	NA	NA
P12	U	U	U	U	U	U	U	U	U	U	NA	NA
P13	33.12	5.2 × 10^4^	33.61	2.7 × 10^4^	32.19	8.67 × 10^3^	35.62	6.48 × 10^3^	33.44	8.6 × 10^3^	23.78	2.94 × 10^6^
P14	U	U	31.73	8.3 × 10^4^	U	U	33.65	2.72 × 10^4^	32.87	2.6 × 10^4^	34.14	4.30 × 10^3^
P15	NA	NA	NA	NA	NA	NA	NA	NA	NA	NA	20.46	2.35 × 10^7^
P16	NA	NA	NA	NA	NA	NA	NA	NA	NA	NA	21.15	1.53 × 10^7^
P17	NA	NA	NA	NA	NA	NA	NA	NA	NA	NA	35.19	2.38 × 10^3^

Cq = threshold cycle; U = Undetectable, refers to negative values obtained from qPCR, indicating that the target pathogen was not detected; NA = Not Available, indicates missing data, either because sampling was not conducted during a specific year or because the genotypes were identified later.

**Table 3:** Pairwise relationship analysis (LRM) performed among the 16 olive genotypes putatively tolerant to *Xfp* and between them and the accessions included in the Di.S.S.P.A. database using GenAlEx v.6.502 software. LRM values of 0.5 indicate complete genetic identity. Moreover, the putative parents of the analyzed genotypes detected using Cervus v.3.0 software are indicated.

Genotype 1	Genotype 2	LRM Value	Putative Parents of Genotype 1	
			First Candidate	Second Candidate
P1_A	P1_B	0.50	-	-
	P3_Sud	0.36	-	-
	P11	0.25	-	-
P1_B	P3_Sud	0.36	-	-
	P11	0.25	-	-
P2	-	-	Alb_Kalinjot_Oval	-
P6	Ciciariello	0.25	IT_Bianca	GRE_Leucocarpa
P7	Pendolino	0.30	IT_Pendolino	-
P8	-	-	IT_Framichele	-
P11	P3_Sud	0.34	-	-
P12	P2	0.29	-	-
	P3_Nord	0.28	-	-
	Leccino_REF	0.27	-	-
P13	P14	0.48	IT_Leccino_REF	Alb_Kalinjot_Oval
	P3_Sud	0.30		
	Alb Kalinjot oval	0.27		
P14	P3_Sud	0.30	IT_Leccino_Lazio	-
	P3_Nord	0.27		
	P7	0.27		
P15	Taggiasca	0.28	-	-
P16	P3_Nord	0.29	ALG_Aayrouni	-
	Ascolana Tenera	0.26		
P17	Cipressino	0.29	-	-

## Data Availability

The raw data supporting the conclusions of this article will be made available by the Authors on request.

## Supplementary Materials

The following supporting information can be downloaded at: https://www.mdpi.com/article/10.3390/plants14020157/s1. Reference [62] is cited in the Supplementary Materials.
